# Eye region surface temperature reflects both energy reserves and circulating glucocorticoids in a wild bird

**DOI:** 10.1038/s41598-018-20240-4

**Published:** 2018-01-30

**Authors:** Paul Jerem, Susanne Jenni-Eiermann, Katherine Herborn, Dorothy McKeegan, Dominic J. McCafferty, Ruedi G. Nager

**Affiliations:** 1 Institute of Biodiversity, Animal Health & Comparative Medicine, University of Glasgow, Glasgow, UK; 2 Swiss Ornithological Institute, Sempach, Switzerland; 3 Institute of Neurobiology, Newcastle University, Newcastle, UK

## Abstract

Body temperature of endotherms shows substantial within- and between-individual variation, but the sources of this variation are not fully understood in wild animals. Variation in body temperature can indicate how individuals cope with their environment via metabolic or stress-induced effects, both of which may relate to depletion of energy reserves. Body condition can reflect heat production through changes to metabolic rate made to protect energy reserves. Additionally, changes in metabolic processes may be mediated by stress-related glucocorticoid secretion, which is associated with altered blood-flow patterns that affect regional body temperatures. Accordingly, both body condition and glucocorticoid secretion should relate to body temperature. We used thermal imaging, a novel non-invasive method of temperature measurement, to investigate relationships between body condition, glucocorticoid secretion and body surface temperature in wild blue tits (*Cyanistes caeruleus*). Individuals with lower body condition had lower eye-region surface temperature in both non-breeding and breeding seasons. Eye-region surface temperature was also negatively correlated with baseline circulating glucocorticoid levels in non-breeding birds. Our results demonstrate that body surface temperature can integrate multiple aspects of physiological state. Consequently, remotely-measured body surface temperature could be used to assess such aspects of physiological state non-invasively in free-living animals at multiple life history stages.

## Introduction

Endotherms maintain a high and relatively constant body temperature. There is, nonetheless, substantial within- and between-individual variation in body temperature among endotherms^1^. Body temperature is a key physiological parameter for understanding thermoregulation, physiology, behaviour and responses to environmental change^2^. Yet, understanding of the factors contributing to body temperature variation in the natural environment remains patchy^3–5^, partly due to difficulties in measuring body temperature in the wild.

Variation in body temperature can reveal how individuals cope with their environment through two main pathways. Firstly, individuals challenged by environmental conditions may show altered hypothalamic-pituitary-adrenal (HPA) axis activity, and glucocorticoid secretion^6^. Activation of the HPA axis can also be associated with activation of the sympathetic-adrenal-medullary (SAM) system^7^. SAM system activity causes rapid release of catecholamines from the adrenal medulla, resulting in a ‘fight or flight’ response. During this response, core body temperature increases^8^, a phenomenon known as stress-induced hyperthermia^9^. Stress-induced hyperthermia is widespread amongst endotherms, and has been detected predominantly as a relatively short-lived (<1 h) response to acute stressors^10^. However, longer term (≥24 h) increases in core body temperature have also been reported in response to social stress^11^, and with repeated stressor application^12–14^.

Secondly, body temperature can reflect metabolic effects, which may also relate to glucocorticoid secretion^6^. For example, chronically lowered core body temperature may be linked to energy reserve depletion, where metabolic rate, and therefore metabolic heat production, is reduced to preserve energy^15^. Simultaneous decreases in metabolic heat production and body temperature have been reported in individuals experiencing poor feeding conditions^16,17^. Also, experimentally elevated circulating glucocorticoid concentrations have been shown both to reduce metabolic rate^18,19^, and increase metabolic rate variability^20^. Consequently, variation in body temperature is predicted to reflect changes in glucocorticoid levels, SAM system activity, energy reserves and metabolic rate.

Assessing body temperature in free-living animals is technically demanding^2^. However, new methods of measuring body surface temperature using thermal imaging^21^ present unique opportunities to determine variation in body temperature (and therefore physiological state), remotely and non-invasively in free living animals. One component of the SAM system response contributing to increased core body temperature during stress-induced hyperthermia is sympathetically-mediated cutaneous vasoconstriction^22^. Arteriovenous anastomoses at the body periphery act as ‘shunts’, permitting passage of blood between arteries and veins on precapillary blood vessels^23^. This redirection of blood away from the body surface (presumably to prepare for ‘fight-or-flight’ by diverting blood to regions with the greatest metabolic need, and/or to minimise potential blood loss from injury to vulnerable areas^22^), lowers body surface temperature^24^. Body surface temperature is also affected by metabolic processes. For an endotherm to maintain a constant core body temperature, heat production must equal heat loss^25^. Accordingly, sustained shifts in the amount of heat transferred to the environment from the body surface can indicate changes to metabolic rate^26^ (although alterations to the insulative capacity of pelage/plumage, may also affect heat loss^27,28^). Heat transfer to the environment is partly mediated by changes in body surface temperature, which alter the temperature gradient between the body surface and the environment^29^. Hence, remotely measured body surface temperature may provide useful information regarding a number of aspects of the physiological state of free-living animals.

In this study, we examined the relationship between body surface temperature, measured using thermal imaging from the eye region (*T*_*eye*_), and physiological state in free-living blue tits (*Cyanistes caeruleus*). Specifically, we tested the hypotheses that lower *T*_*eye*_ is associated with lower body condition (indicating reduced energy reserves), and higher circulating glucocorticoid concentrations (indicating HPA axis activity).

## Results

*T*_*eye*_ ranged from 25.2–33.6 °C (29.6 ± 0.09 °C, n = 372 observations from 14 individuals) in the breeding birds, and was higher than the *T*_*eye*_ of 26.5–31.2 °C (28.7 ± 0.23 °C, n = 31) recorded in the overwintering birds (t = 1.98, df = 43, p = 0.05). Additionally, *T*_*eye*_ was lower when breeding birds were about to enter the nest box (‘In’, n = 178) than when they left (‘Out’ n = 189), or when they approached, but did not enter (‘No Entry/Exit’ n = 5) (Table 1). Compared to winter *T*_*eye*_ measurements, mean individual *T*_*eye*_ measured during ‘Out’ events was higher (t = 2.70, df = 42, p = 0.01), whereas *T*_*eye*_ measured during ‘In’ events did not differ (t = 0.68, df = 43, p = 0.50).

**Table 1:** Summary of statistical models relating body condition index with *T*_*eye*_, accounting for air temperature and humidity in (a) winter (GLM model), and (b) breeding season (GLMM model); *r* is the parameter effect size. In the breeding season whether birds were entering (Event Type = ‘In’, reference value in GLMM), leaving (Event Type = ‘Out’), or approaching the nest box without entering (Event Type = ‘No Entry/Exit’) was also considered (see Methods).

	Response Variable: Baseline Eye Region Temperature (*T*_*eye*_)				
	Fixed Effects	Estimate ± 95% CI	t-value	p-value	*r*
**a**.	Intercept	26.44 ± 1.05	49.24	<0.0001	
	Body Condition Index	0.69 ± 0.66	2.05	0.05	0.37
	Air Temperature	0.29 ± 0.13	4.45	0.0001	0.65
	Relative Humidity	−0.02 ± 0.02	1.61	0.12	0.30
	**Model adjusted R**^**2**^ = **0.47**				
**b**.	Intercept	25.87 ± 1.39	36.52	<0.0001	
	Body Condition Index	0.86 ± 0.69	2.43	0.028	0.09
	Air Temperature	0.23 ± 0.09	4.76	<0.0001	0.17
	Relative Humidity	−0.02 ± 0.02	1.14	0.26	0.04
	Event Type (Out)	1.01 ± 0.18	11.19	<0.0001	
	Event Type (No Entry/Exit)	1.24 ± 0.78	3.12	0.002	
	**Random Effect**	**Variance**	**SD**	**p-value**	
	Bird ID	0.77	0.88	<0.0001	
	**Model marginal R**^**2**^ = **0.35**				

**Figure 1:**
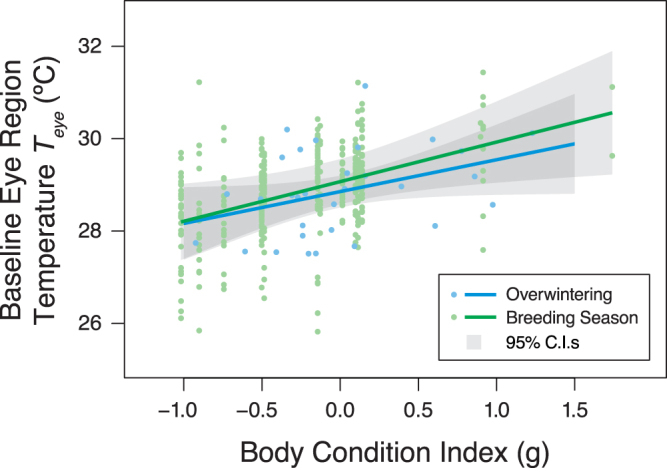
Model predictions of relationships between baseline eye region temperature (*T*_*eye*_) and body condition index. Zero represents mean condition, with positive and negative values indicating body mass above or below average, respectively, for a given wing length. For the breeding season data, the model prediction is conditional on event type being when birds were entering the nest box (event type ‘In’), as opposed to exiting (event type ‘Out’) – see methods. This approach was chosen as *T*_*eye*_ measured during ‘In’ events is more comparable to that of overwintering birds, than *T*_*eye*_ measured during ‘Out’ events. ‘In’ events share similar activity levels with overwintering birds entering the trap, and do not involve the residual warming effect of the nest box microclimate on *T*_*eye*_ during ‘Out’ events (Table 2b and see Discussion).

**Figure 2:**
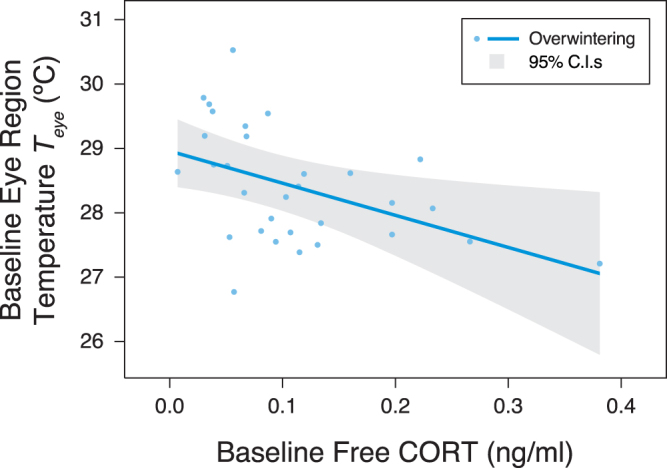
Model prediction of relationship between baseline eye region temperature and baseline free CORT.

*T*_*eye*_ increased with body condition in both seasons (Fig. 1) after accounting for a positive effect of *T*_*a*_ on *T*_*eye*_ (Table 1). While the winter and breeding season body condition models differ structurally (and so cannot be directly compared), confidence intervals for the body condition index parameter estimates largely overlap (Table 1), indicating similar relationships. The relationship between *T*_*eye*_ and CORT was only investigated in winter. Baseline free CORT concentrations varied from 0.007–0.381 ng/ml (0.11 ± 0.02 ng/ml, n = 31), while baseline total CORT concentrations varied between 0.999–18.61 ng/ml (8.29 ± 0.65 ng/ml, n = 31). Of the total CORT concentration, 1.41 ± 0.18% was available as free CORT. Sampling latency was unrelated to baseline free or total CORT (free CORT *F*_1,29_ = 0.002, *p* = 0.97, total CORT *F*_1,29_ = 0.93, p = 0.34). After controlling for ambient conditions, *T*_*eye*_ during winter was negatively associated with baseline free CORT concentration (Table 2a, Fig. 2), but was not associated with baseline total CORT concentration (Table 2b). Neither baseline free nor total CORT were related to body condition index (free CORT *F*_1,29_ = 0.40, *p* = 0.53, total CORT *F*_1,29_ = 2.19, p = 0.15).

**Table 2:** Summary of GLM models relating air temperature, humidity and (a) baseline free CORT or (b) baseline total CORT with *T*_*eye*_ in winter; *r* is the parameter effect size.

	Response Variable: Baseline Eye Region Temperature (*T*_*eye*_)				
	Fixed Effects	Estimate ± 95% CI	t-value	p-value	*r*
**a**.	Intercept	30.70 ± 2.79	21.50	<0.0001	
	Baseline free CORT	−4.99 ± 3.84	2.55	0.017	0.44
	Air temperature	0.19 ± 0.13	2.90	0.007	0.49
	Relative Humidity	−0.034 ± 0.022	3.09	0.005	0.51
	**Model adjusted R**^**2**^ = **0.56**				
**b**.	Intercept	28.71 ± 0.41	135.86	<0.0001	
	Baseline total CORT	0.028 ± 0.098	0.55	0.586	0.11
	Air Temperature	0.24 ± 0.14	3.25	0.003	0.53
	Relative Humidity	−0.025 ± 0.022	2.18	0.008	0.34
	**Model adjusted R**^**2**^ = **0.48**

## Discussion

Baseline eye region surface temperature (*T*_*eye*_) was related both to body condition and baseline glucocorticoid levels in undisturbed wild blue tits. This suggests that individual differences in body surface temperature can reflect physiological state. Reduced metabolic rate in individuals with depleted energy reserves, and/or stress-related vasoconstriction may have brought about the observed between-individual variation in *T*_*eye*_.

The level of variation in *T*_*eye*_ observed in this study was comparable in extent to that previously reported for both surface and subcutaneous measures of body temperature in parids^4^. *T*_*eye*_ was influenced by ambient conditions, with *T*_*a*_ and relative humidity explaining a significant proportion of variation. The positive relationship between *T*_*eye*_ and *T*_*a*_ is consistent with other studies showing surface temperature in small birds to be sensitive to environmental conditions^3,4,30,31^. Despite this, high repeatabilities of surface temperature (>0.7) have been reported for periods up to 40 min in great tits (*Parus major*)^4^. Yet, even when accounting for these environmental influences on *T*_*eye*_, there remained between-individual variation in *T*_*eye*_ that was related to measures of physiological state.

In the breeding season, *T*_*eye*_ was also affected by event type, with higher values of *T*_*eye*_ being recorded during ‘Out’ and ‘No Entry/Exit’ events. As mean individual *T*_*eye*_ from ‘In’ events did not differ from *T*_*eye*_ measured in winter, the overall difference in *T*_*eye*_ between seasons must relate to the presence of *T*_*eye*_ measurements from ‘Out’ and ‘No Entry/Exit’ events in the breeding season data. Differences in *T*_*eye*_ between event types in the breeding birds most likely result from a composite of behavioural differences, and the warming effect of having spent time in the nest box microclimate. While birds entering the nest box were rarely stationary, birds leaving the nest box often paused (presumably to check for potential hazards) before exiting. Similarly, birds which entered the field of view of the camera, but did not enter or exit also tended to spend more time stationary than birds entering the nest box. As negative measurement error during thermal imaging of small passerines results predominantly from motion blur^21^, values of *T*_*eye*_ tend to increase with the inactivity of the subject. However, it also remains possible that elevated *T*_*eye*_ in ‘No Entry/Exit’ events was a statistical artefact relating to their limited number.

*T*_*eye*_ was positively and similarly related to body condition at two stages of the annual cycle. Body condition can be linked to body surface temperature as a result of variation in the insulative capacity of plumage. Anatomical modulation of plumage insulation capacity post-moult is thought to be relatively limited^32^. Nevertheless, parasite-induced changes to feather mass have been shown to affect heat loss to the environment, metabolic rate, and body condition in feral rock doves (*Columba livia*)^28^. Reduced feather mass was associated with increased heat loss and metabolic rate, and a continuous decline in body condition over the course of the nine-month study. The authors attributed these outcomes to the heightened energy cost of maintaining core body temperature in birds with lower feather mass, as core body temperature was similar across all birds sampled regardless of plumage insulation capacity. Increased heat loss caused by reduced insulation occurs due to increased body temperature at the interface with the environment, which steepens the ambient temperature gradient. As a consequence, it seems doubtful that reduced plumage insulation capacity was an important factor in our study, as we found lower body condition was associated with lower body surface temperature.

Instead, the observed relationship between *T*_*eye*_ and body condition may indicate a response to environmental challenge. For example, reductions in metabolic rate, and resulting heat production, can occur in individuals experiencing poor feeding conditions^16^. At low ambient temperatures, reducing body temperature can diminish the temperature gradient by which heat is lost from the body, offsetting thermoregulatory costs^5^. Both processes can provide energy savings for birds with limited reserves. Environmental challenges may not only reduce body condition and induce changes to metabolic rate, but can also lead to physiological stress, and changes in HPA activity^33^. Equally, as HPA activity directly affects metabolic rate^18,19^, circulating glucocorticoid levels may act as mediators of metabolic responses to environmental challenges. We found that after accounting for ambient conditions, *T*_*eye*_ was also positively related to free, but not total baseline CORT levels. If free CORT concentrations, body condition and *T*_*eye*_ were all indicative of physiological stress in this study, then a relationship between body condition and free CORT might also have been expected, in addition to those observed between *T*_*eye*_ and body condition, and *T*_*eye*_ and free CORT. We did not find such an association, although this may be a result of the restricted sample sizes achieved in this study, when compared with others reporting relationships between glucocorticoid levels and body condition in birds^34–36^. It is also possible that physiological stress and energy reserves are independently linked to *T*_*eye*_. Alternatively, a relationship between *T*_*eye*_ and baseline free CORT could be mediated by locomotor activity. Increased CORT levels generally stimulate increased locomotor activity^37^, which would be expected to increase heat production, and therefore surface temperature. However, we found that higher levels of baseline free CORT were associated with lower *T*_*eye*_, indicating that locomotor activity could not have been responsible for the observed relationship. Also, variation in locomotor activity prior to entering the trap (i.e. flight distance/duration) could have influenced *T*_*eye*_, potentially confounding relationships with CORT and/or body condition. The density of the forest in which the fieldwork took place meant it was not possible to meaningfully assess flight distance or duration. Nonetheless, our ability to detect relationships between *T*_*eye*_ and CORT/body condition, without taking flight distance/duration into account, suggests their confounding effects were limited.

Free baseline CORT levels observed in this study were within the range of those found elsewhere in small passerines^38–40^. Despite this, the free proportion of total CORT we report (1.4 ± 1.0%) appears low when compared to the commonly cited 5–10%^41^. Low proportions of free CORT to total CORT could be a result of two factors. Firstly, tissue glucocorticoid receptors may respond differently between groups or species, perhaps meaning fewer glucocorticoids are necessary to generate the necessary response^42^. Secondly, relatively high concentrations of total CORT may mean that lower proportions of free CORT are sufficient to trigger desired responses. In this context, it is interesting to note that total CORT levels observed in this study were higher than the majority of values reported for blue tits^34,38,43,44^.

As sampling latency was unrelated to CORT levels, our values most likely represent true baselines, and were not overestimated due to the acute stress of trapping and handling. *T*_*eye*_ in free-living blue tits also responds to acute stress, but on a shorter time scale than CORT^21^. *T*_*eye*_ could potentially be affected by the acute stress of entering the trap. Yet, this is unlikely as the traps were left in place at least one month prior to sampling to encourage habituation. Nevertheless, potential acute stressors experienced shortly before entry into the trap, and/or surface temperature responses to anticipation and consumption of food^45^ could mask any relationship between *T*_*eye*_ and baseline glucocorticoids/body condition. That we found the predicted relationship suggests our results are conservative in both respects. Furthermore, when using baited traps, there is the possibility of sampling bias relating to individual differences in personality^46^. Shy, neophobic individuals are less likely to enter traps of the kind used in this study, potentially resulting in a sampling bias towards bold individuals. However, shy individuals have been shown to exhibit higher total CORT concentrations^47,48^. Therefore, given that the mean total CORT concentrations we report were comparatively high^34,38,43,44^, a bias towards bold individuals in our sample appears unlikely.

Whether free or total CORT concentrations play a greater biological role remains an open question^6^. If only the free proportion of total CORT is physiologically active^49^, and HPA axis activity is linked to stress-induced hyperthermia, then only a relationship between *T*_*eye*_ and free CORT would be expected, as found in this study. A further possibility could involve alternative mechanisms predicted to be responsible for differential changes in free and total CORT concentrations. Subjecting white crowned sparrows (*Zonotrichia leucophrys*) to 23 h of fasting resulted in increased free CORT concentrations, but not total CORT^50^. This was attributed to a drop in CBG binding, related either to CORT release inducing CBG breakdown, or to stress-induced elevations of CORT being overridden by the daily peak in CORT secretion. Both processes could potentially lead to differing relationships between *T*_*eye*_ and free/total CORT, although neither seem likely here. If free CORT concentrations increased with CORT secretion, a correlation would be expected between total and free CORT. This was not the case in this study (Spearman’s ρ = 0.29, p = 0.11). Equally, the daily peak in CORT secretion would be expected to occur at/towards the end of the inactive period^51^. As all sampling during this study took place within the active period, it seems unlikely that the daily peak would have influenced our measurements of CORT. Finally, it may also be possible that while *T*_*eye*_ and free CORT appear associated, they are actually only exhibiting synchronized natural variation with circadian rhythms. However, if this were the case, a clear relationship between free CORT and time of day would be expected, but this was not detected (GLM; time of day *F*_1,29_ = 0.0003, p = 0.99).

Our study showed that variation in body surface temperature and body condition were similarly associated during both non-breeding and breeding seasons, and that body surface temperature was related to baseline circulating glucocorticoid levels in non-breeding birds. Lower body surface temperature in individuals with lower body condition could relate to reductions in metabolic rate made to conserve energy, or it could be linked with stress responses to poor food intake. The two explanations are not mutually exclusive, but the lack of relationship between CORT and body condition demonstrates that body surface temperature can integrate multiple aspects of physiological state. Hence, remote measurement of body surface temperature using thermal imaging could provide a novel, non-invasive means of assessing the physiological state of free-living animals. Our results highlight important relationships between body temperature and underlying physiology for which mechanistic understanding remains incomplete. Investigations targeting the processes linking body condition, glucocorticoid secretion and body temperature would be of particular value in this respect. Moreover, exploration of relationships between body surface temperature and further biomarkers of physiological state (e.g. plasma protein concentrations, cholinesterase activity, heterophil/lymphocyte ratio, telomere lengths) may not only help distinguish effects of varying energy reserves and physiological stress, but could also yield opportunities to infer additional parameters non-invasively.

## Methods

### Fieldwork

Data were collected from overwintering and breeding blue tits occupying oak woodland on the eastern shore of Loch Lomond (56.13°N, 4.13°W). Birds were recorded on thermal videos either entering walk-in box traps (winter), or their nest box (breeding season) (see Thermal Imaging section below). In winter, we trapped 31 blue tits. Once a single bird entered the trap it was allowed to feed undisturbed while thermal videos were recorded. The trap was then closed, the bird retrieved and a blood sample taken to measure baseline circulating total and free corticosterone concentrations (see Glucocorticoid Assays section below). During the breeding season, thermal videos were recorded of 14 birds as they repeatedly visited their brood, over a period of 30 min on day 13 after hatching. Birds sampled during the breeding season were not part of the winter study. All fieldwork was approved by the UK Home Office, and carried out in accordance with the Animals (Scientific Procedures) Act 1986.

**Figure 3:**
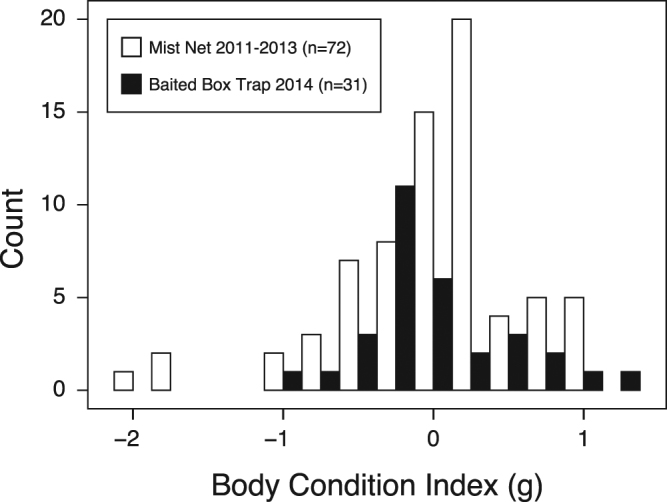
Body condition index distributions for birds caught using baited box traps during the winter of 2014 in this study, and mist-netted birds caught at the same site, during the same time of year (first two weeks in March) in three previous years (2011, 2012, 2013). A body condition index of zero represents mean condition, with positive and negative values indicating body mass above or below average, respectively, for a given wing length.

During the winter of 2013/14, walk-in box traps^21^ were installed at four locations (inter-trap distance 0.8 ± 0.2 km) across the study site. To habituate visiting birds to the recording set-up and avoid sampling bias towards bold individuals, the traps were continuously baited with granulated peanuts for >1 month before sampling. The distribution of individual body condition index (calculated as the residuals of an ordinary least squares regression of mass against wing chord cubed^52^) from birds sampled during this study was not significantly different to that from mist-netted birds caught at the same site during the same time of year (first two weeks in March), in three previous years (2011–2013) (Kolmogorov-Smirnov test, D = 0.224, p = 0.225, Fig. 3). This indicated no detectable effect of sampling bias in terms of body condition relating to the use of baited traps in this study, and notably no bias towards birds with particularly low energy reserves.

The 31 blue tits caught in the winter were trapped during 10–13 March 2014 between 08:18 and 16:52 (mean sampling time of day 12:50 ± 32 min). The end of winter was selected as the period in winter when the greatest differences in individual state would be expected, and when relationships between body temperature and state would be most detectable. The relatively short sampling period was chosen to minimise (and control for) environmental variation, as much as possible, as environmental conditions can influence body temperature and physiological processes affecting state^53,54^. Daytime sampling was chosen to minimise the effect of circadian changes in body temperature^55^.

Single birds entering the trap were filmed using a thermal imaging camera (see below), while feeding undisturbed for an average of 4.2 ± 0.16 s, before the trap was closed by the experimenter (using a fishing line, from a concealed position). The 4.2 s duration is sufficient to obtain a representative measurement of temperature for that bird^21^. The bird was then retrieved by hand 17.4 ± 0.75 s after trap closure. Approximately 30 µl of blood was sampled from the jugular vein by venipuncture within 112.3 ± 3.2 s of trap closure, and immediately placed on ice. Blood samples were subsequently separated into plasma and red blood cells by centrifugation (10 min at 2000 rpm), and the plasma used to measure baseline circulating corticosterone (CORT) concentration. Before the birds were released, we identified sex from plumage characteristics^56^, and measured body mass (to the nearest 0.1 g), and maximal wing chord (to the nearest 0.5 mm). Each bird was sampled only once. Repeated trapping/sampling was avoided by establishing the identity of ringed birds from their colour ring combination, either before shutting the trap, or during handling. Trapped unringed birds were fitted with a uniquely identifying colour ring combination after sampling, prior to being set free.

During the breeding season, breeding parents were filmed while entering or exiting their nest box using a thermal imaging camera mounted in front of the nest box (Schwegler 1B, with 32 mm entrance hole). To habituate the birds to the presence of the camera, a dummy camera was installed at each nest ≥7 days prior to filming, and left in place. The dummy camera was replaced with the actual camera immediately prior to filming on day 13 after hatching (day of hatching = day 0; filming took place during 5–16 June 2015, between 10:09 and 20:31, mean sampling time 13:05 ± 8 min). Daytime sampling was again chosen to minimise the effect of circadian changes in body temperature. Once the camera was installed, thermal video was recorded for 45 minutes. To avoid acute stress-related changes in body temperature resulting from disturbance associated with installation, only body temperature measures from video recorded after the initial 15 minutes were included in the analyses. To distinguish between the two parents at each nest, we caught at least one of them (14 individuals breeding in 12 randomly selected nests) on day 8–9 after hatching (between 01–11 June 2015), and fitted an RFID tag (125 kHz, 2.3 mm, EM4102 Bird Tag, IB Technology Glenfield, Leicestershire) mounted on a leg ring. Parents were then identified on the thermal videos using a combination of the records of the RFID tag logger mounted in the nest box entrance (Nature Counters, Maidstone, Kent; IB Technology Glenfield, Leicestershire; Francis Scientific Instruments, Ltd., Huntingdon, Cambridgeshire; University of Glasgow Bioelectronics Dept., Glasgow), and distinguishing features (e.g. leg rings) visible in the thermal images. During RFID fitting, sex was identified, and body mass/wing chord length taken for calculation of body condition index using the same techniques described above for overwintering birds.

Ambient temperature (*T*_*a*_) was recorded simultaneously to the thermal imaging using a Tinytag Talk 2 Temperature Logger (Gemini Data Loggers UK, Chichester, West Sussex) mounted on the box trap (winter) or nest box (breeding season). In winter, relative humidity was recorded every 30 min (Minimet, Skye Instruments, Wales) at the centre of the study site. During the breeding season, a weather station malfunction meant that humidity data were instead obtained from the MIDAS MET Office weather station at Bishopton (approximately 25 km from the study site; 55.91°N, −4.53°W). For a short period during the breeding season where data were available from both weather stations, the humidity data were strongly correlated (GLM: *F*_1,15_ = 35.68, p < 0.0001, R^2^ = 0.70). This confirmed that substituting the local weather station data with the MIDAS data was appropriate.

### Thermal imaging

During all thermal imaging, the camera (FLIR A65, f = 25 mm, spatial resolution 0.68 mrad, FLIR Systems, Wilsonville, Oregon) was mounted at a distance of 50 cm so birds were recorded passing through the camera’s field of view and within the camera’s zone of focus, either within the trap, or as they entered and left the nest box (detailed description of trapping/filming setup available in^21^). We measured body surface temperature from the region of exposed skin around the eye, an area comprised of approximately 230 pixels. Maximum eye region temperatures (*T*_*eye*_) were extracted from the thermal video, as the highest temperature measured from the eye region is assumed to be the most accurate^21^. When thermal imaging small passerines, where the majority of the body is insulated by feathers, the exposed eye region is surrounded by cooler integument. Motion blur (which occurs when activity is too rapid to be captured by the camera’s frame rate) causes data from the small warm eye area of the image to be confounded with that of larger neighbouring cooler areas, resulting in an underestimation of *T*_*eye*_. In contrast, overestimation of *T*_*eye*_ requires energy input. This was avoided or accounted for by eliminating/assessing exposure to solar radiation (see below). Accordingly, the maximum temperature measured from the eye region was always likely to be the most accurate measurement recorded. The eye region was chosen as the region of interest, as the periorbital skin is the only area of the body surface where heat transfer to the environment (and therefore surface temperature) is not modulated by insulating feathers or leg scales^29,57^. Also, the legs play a substantial thermoregulatory role in birds^58^. Thermoregulatory manipulation of heat transfer to the environment from the legs is likely to obscure relationships between body surface temperature and energy reserves/physiological state. As a consequence, the legs were not considered a suitable site for assessing such relationships.

Baseline *T*_*eye*_ was defined as the maximum temperature measured from the eye region of an individual while (i) feeding undisturbed within the trap (winter), (ii) during entry/exit from the nest box (breeding season) and (iii) during periods where the bird was in the camera’s field of view, if it did not enter or leave the nest box (breeding season). The type of event from which *T*_*eye*_ was measured during the breeding season could influence the value of *T*_*eye*_ due to the microclimate of the nest box (exit) or as a result of exercise during the preceding foraging trip (entry). Therefore, event type was noted and included in the breeding season analysis. Visits to the nest box were defined as ‘In’ when an individual entered the nest box, ‘Out’ when an individual left the nest box, and ‘No Entry/Exit’ when an individual entered the field of view of the camera, but did not enter or exit the nest box. Body temperature may also be influenced by solar radiation^59^. While the box traps were oriented such that birds within were shielded from the sun, the breeding birds were not always completely in the shade. Therefore, the presence/absence of direct solar radiation falling on the nest box during thermal imaging was recorded as a categorical measure during the breeding season.

Accurate absolute temperatures can be estimated from thermal images by the inclusion of an object of known temperature and emissivity within the field of view, against which the temperature measured from the bird’s surface by the thermal imaging camera can be calibrated. To achieve this, a thermistor probe coated in black insulation tape (Tesa UK, Milton Keynes, Buckinghamshire) was installed onto the front of the trap (winter) or the nest box perch (breeding season), and connected to the temperature logger described above. The logger was set to record at 1 s intervals, and the resulting temperature data used to calibrate the individual frames from which *T*_*eye*_ was extracted.

### Glucocorticoid assays

Total CORT levels were assessed using a commercial ELISA kit (Enzo Life Sciences, Switzerland), following the manufacturer’s instructions. This ELISA kit had been validated for the European blackbird (*Turdus merula*), and the great tit (*Parus major*), a close relative of the blue tit^60^. One reading was below the detection limit (1 ng/ml), so was set to 0.999 ng/ml. Corticosteroid-binding globulin (CBG) affinity and capacity were assessed, and free CORT levels estimated following the methods described by Breuner *et al*.^61^. For individual birds, CBG was estimated using 20 nM [^3^H] corticosterone. Maximum site binding capacity (*Bmax)*, and the dissociation constant (*Kd)* from the saturation analysis were calculated using iterative, least-squares curve-fitting (GraphPad Prism, GraphPad Software, US) to fit untransformed data to a single site binding hyperbola (y = Bmax*x/(*Kd* + x)). *Kd* was 2.58 nM, whilst *Bmax* was 321.9 ± 13.27 nM (Supplementary Information Figs 1 and 2). The intra- and inter-assay coefficients of variation were 11.25% and 3.85%, respectively.

### Statistical analyses

All statistical analyses were performed using R v3.1.2^62^. Individual *T*_*eye*_ measurements were compared between seasons and event types using two sample t-tests. Relationships between *T*_*eye*_ (as the response variable) and body condition index, free CORT and total CORT during winter were analysed using separate multivariate general linear models (GLM) for each focal explanatory variable. Associations between free/total CORT (as the response variables) and body condition index were tested for using univariate GLMs. As repeated measures of *T*_*eye*_ were taken from breeding birds, the relationship between *T*_*eye*_ (response variable), and body condition index during the breeding season was analysed using a multivariate general linear mixed model (GLMM – ‘lme4’ package v1.1–12^63^) with Bird ID specified as a random effect.

CORT shows circadian rhythms, responds to environmental conditions, and can differ both between sexes^6^, and with previous experience of trapping/handling^64^. Also, body temperature fluctuates with a circadian rhythm, is modulated by air temperature (*T*_*a*_)^53^, humidity^54^, and solar radiation^65^, and may differ between sexes^66^. Therefore, time of day, *T*_*a*_, humidity, presence/absence of direct sunlight, sex and whether birds were entering or exiting their nest box (event type) were considered as potential confounding explanatory variables. Potential confounding explanatory variables were included in the full multivariate models only if they were significantly associated with *T*_*eye*_ in preliminary univariate tests (critical p = 0.1). In these preliminary analyses, *T*_*eye*_ differed between event types, so event type was included in the breeding season analysis. However, *T*_*eye*_ did not differ between sexes (*F*_1,29_ = 2.57, p = 0.12), or with the presence/absence of direct sunlight (*t* = 0.81, p = 0.43). Therefore, these variables were not included in the full models. All other potential confounding explanatory variables were related to *T*_*eye*_ in the univariate tests (p < 0.017). Time of day was correlated with *T*_*a*_, so including both variables would result in a collinearity issue that would prevent discrimination between diurnal changes in *T*_*eye*_ and environmental effects on *T*_*eye*_. As our primary concern was to account for environmental influences, and we restricted measurement to a relatively short period of the day over which diurnal changes are minimal, we included *T*_*a*_, but not time of day, in the multivariate models.

Significance of explanatory variables (critical two-tailed p < 0.05) was determined via backwards-stepwise model selection using *drop1*. P-values for fixed/random effects in the GLMM were calculated using *lmerTest/rand* (‘lmerTest’ package v1.0^67^). Effect size *r* for parameters in the final models, and *R*^2^ for the GLMM were calculated using equations specified by Nakagawa & Cuthill^68^, and Nakagawa & Schielzeth^69^, respectively. Variance inflation factors (*vif*, ‘car’ package v2.0–22^70^) calculated post-hoc for explanatory variables included in the GLMs suggested no issue with collinearity. All model assumptions were met. Throughout the results we report means ± standard error.

### Data Accessibility

 Data available from the Dryad Digital Repository: 10.5061/dryad.1jg00.

## Electronic supplementary material

Supplementary Information
