# Supplementary material for: Eye region surface temperature reflects both energy reserves and circulating glucocorticoids in a wild bird
Source: Sci Rep. 2018 Jan 30;8:1907. doi: 10.1038/s41598-018-20240-4 (PMC5789886; doi:10.1038/s41598-018-20240-4)
Supplement: Supplementary file 1 — Supplementary Information [file 41598_2018_20240_MOESM1_ESM.pdf]

Eye region surface temperature reflects both energy reserves and circulating glucocorticoids in a wild bird.

Paul Jerem[1*], Susanne Jenni-Eiermann[2], Katherine Herborn[1,3], Dorothy McKeegan[1], Dominic J. McCafferty[1] & Ruedi G. Nager[1]

1. Institute of Biodiversity, Animal Health & Comparative Medicine, University of Glasgow, Glasgow, UK.

2. Swiss Ornithological Institute, Sempach, Switzerland.

3. Institute of Neurobiology, Newcastle University, Newcastle, UK.

*paul@pauljerem.com

Supplementary Information

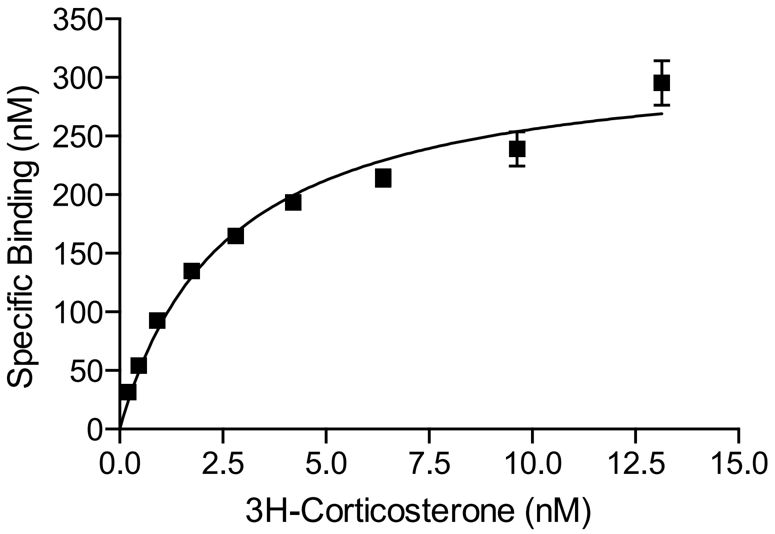

Supporting Information Figure 1 Specific binding of [3H] corticosterone to blue tit plasma in relation to increasing concentrations of radiolabeled corticosterone. Solid squares represent means ± standard error.

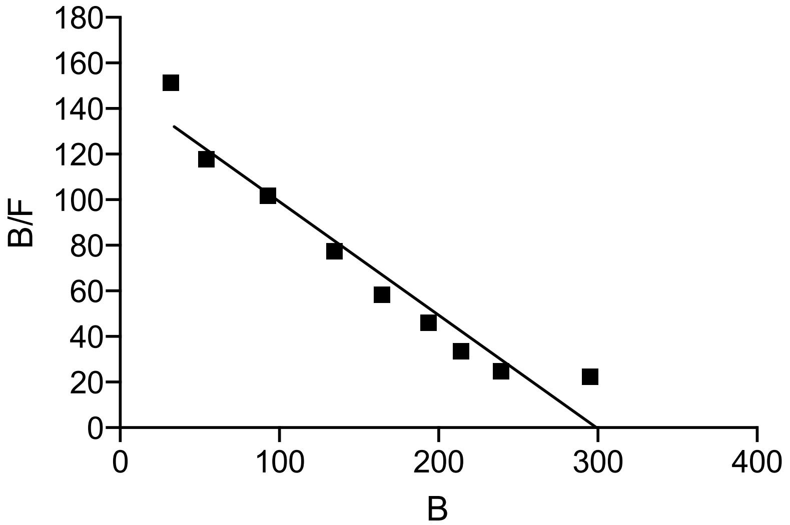

Supporting Information Figure 2 Scatchard-Rosenthal re-plot of Figure 4-2, where F = free, and B = bound [3H] corticosterone fraction.
